# microRNA-320/RUNX2 axis regulates adipocytic differentiation of human mesenchymal (skeletal) stem cells

**DOI:** 10.1038/cddis.2014.462

**Published:** 2014-10-30

**Authors:** D Hamam, D Ali, R Vishnubalaji, R Hamam, M Al-Nbaheen, L Chen, M Kassem, A Aldahmash, N M Alajez

**Affiliations:** 1 Stem Cell Unit, Department of Anatomy, College of Medicine, King Saud University, Riyadh, Saudi Arabia; 2 KMEB, Department of Endocrinology, University of Southern Denmark, Odense, Denmark

## Abstract

The molecular mechanisms promoting lineage-specific commitment of human mesenchymal (skeletal or stromal) stem cells (hMSCs) into adipocytes (ADs) are not fully understood. Thus, we performed global microRNA (miRNA) and gene expression profiling during adipocytic differentiation of hMSC, and utilized bioinformatics as well as functional and biochemical assays, and identified several novel miRNAs differentially expressed during adipogenesis. Among these, miR-320 family (miR-320a, 320b, 320c, 320d and 320e) were ~2.2–3.0-fold upregulated. Overexpression of miR-320c in hMSC enhanced adipocytic differentiation and accelerated formation of mature ADs in *ex vivo* cultures. Integrated analysis of bioinformatics and global gene expression profiling in miR-320c overexpressing cells and during adipocytic differentiation of hMSC identified several biologically relevant gene targets for miR-320c including RUNX2, MIB1 (mindbomb E3 ubiquitin protein ligase 1), PAX6 (paired box 6), YWHAH and ZWILCH. siRNA-mediated silencing of those genes enhanced adipocytic differentiation of hMSC, thus corroborating an important role for those genes in miR-320c-mediated adipogenesis. Concordant with that, lentiviral-mediated stable expression of miR-320c at physiological levels (~1.5-fold) promoted adipocytic and suppressed osteogenic differentiation of hMSC. Luciferase assay validated RUNX2 (Runt-related transcription factor 2) as a bona fide target for miR-320 family. Therefore, our data suggest miR-320 family as possible molecular switch promoting adipocytic differentiation of hMSC. Targeting miR-320 may have therapeutic potential *in vivo* through regulation of bone marrow adipogenesis.

Bone marrow fat is increasingly recognized as an important component of the bone marrow microenvironment with potential role in regulating bone formation, hematopoiesis and the whole body's energy metabolism.^1,2^ During aging and in a number of skeletal diseases, an inverse relationship between bone marrow trabecular bone mass and fat mass has been reported, suggesting a common regulatory genetic program.^3, 4, 5, 6^ Based on a large number of *in vitro* studies, bone marrow adipocytes (ADs) and osteoblasts originate from a common progenitor cells within the bone marrow stroma known as mesenchymal (skeletal or stromal) stem cells (MSCs).^7^ It is thus envisaged that controlling MSC fate into osteoblasts or AD can be a target for intervention with the aim of enhancing bone formation in bone loss disorders.^8^ To achieve this goal, molecular mechanisms controlling MSC commitment to ADs *versus* osteoblasts need to be identified.

MicroRNAs (miRNAs) are double-stranded noncoding RNA molecules of ~22 nucleotides that function as post-transcriptional regulators of gene expression and are found in a wide variety of organisms, from plants, insects to humans.^9,10^ miRNAs have been identified to affect multiple biological functions including stem cell differentiation, neurogenesis, hematopoiesis, immune response, skeletal and cardiac muscle development.^11, 12, 13, 14, 15, 16, 17^ Several previous studies have identified a number of miRNAs as important regulators of MSC differentiation into osteoblasts (for review, see Taipaleenmaki *et al.*,^18^, Eskildsen *et al.*^19^ and Zeng *et al.*^20^) and chondrocytes.^21^ On the other hand, few studies have examined miRNA regulation of MSC differentiation into ADs.^22,23^ In the present study, we carried out a comprehensive analysis of miRNA expression profiling and bioinformatics analyses of human MSC (hMSC) during *in vitro* AD differentiation. We identified several novel pro-adipogenic miRNAs, and found that miR-320 to be an important regulator of adipocytic differentiation of hMSC.

## Results

### Identification of differentially expressed miRNAs during adipocytic differentiation of hMSCs

**Figure 1:**
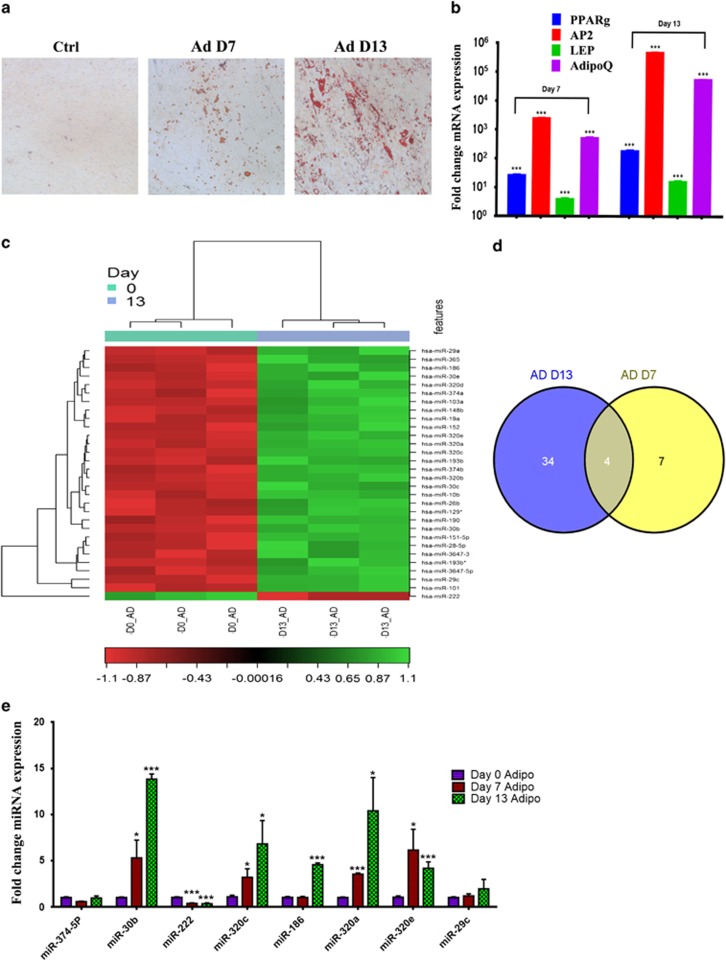
miRNA expression profiling during adipocyte (AD) differentiation of hMSC. hMSC-TERT cells were induced to AD differentiation. (**a**) Oil Red O staining of lipid-filled mature ADs on days 7 and 13. (**b**) qRT-PCR analysis of AD marker genes (peroxisome proliferator-activated receptor*-γ*, *PPARγ*; AD protein 2, *AP2*; leptin, *LEP*; adiponectin, *AdipoQ*). Gene expression was normalized to GAPDH and *β*-actin. Data are presented as mean±S.E. of fold changes compared with non-induced controls, *n*=6 from two independent experiments. ***P<0.0005 between non-induced and induced samples. hMSC were induced to AD differentiation and on days 0, 7 and 13. miRNA expression profiling was done using the miRCURY LNA miRNA Array (6th GEN). (**c**) Heat map and unsupervised hierarchical clustering were performed on the top 30 miRNAs differentially expressed on AD day 13 (D13) *versus* AD D0; the color scale illustrates the relative expression level of miRNAs (log2). Red color represents an expression level below the reference channel, and green color represents expression higher than the reference. (**d**) Venn diagram depicting the overlap in miRNAs that were differentially expressed on AD D13 and AD D7. (**e**) Validation of selected miRNAs identified in **c** using Taqman miRNA qRT-PCR. Data are presented as mean±S.E., *n*=6. *P<0.05, ***P<0.0005

Using standard AD-induction medium (AIM), hMSC differentiated readily into mature lipid-filled ADs as demonstrated by positive staining for Oil Red O (Figure 1a) and increased expression of several AD-specific genes (Figure 1b). Global miRNA expression profiling carried out on AD-differentiated hMSC revealed 38 miRNAs to be differentially expressed on day 13 compared with day 0 (*P*<0.05; Figure 1c and Table 1). Eleven miRNAs were found to be differentially expressed on day 7 compared with day 0 (*P*<0.05; Figure 1d and Supplementary Table 1). The expression levels of selected group of miRNAs identified in the microarray experiment, miR−374−5p, −30b, −222, −320c, −186, −320a,−320e and −29c, were validated using quantitative real-time reverse transcription PCR (qRT-PCR) that confirmed the microarray results and showed upregulation of miR-30b, miR-320 family (320a/320c/ 320e) on day 7 post-AD differentiation induction and further increase in expression levels of the same miRNAs in addition to miR-186 on day 13 (Figure 1e). miR-222 was found to be downregulated on day 13 (Figure 1e). Among the identified miRNAs, several members of the miR-320 (miR-320a, 320b, 320c, 320d and 320e) family were differentially expressed and were chosen for further investigation, as they have not previously been implicated in regulating the adipocytic differentiation of MSCs.

### Overexpression of miR-320c and miR-30b promote adipocytic differentiation of hMSCs

**Figure 2:**
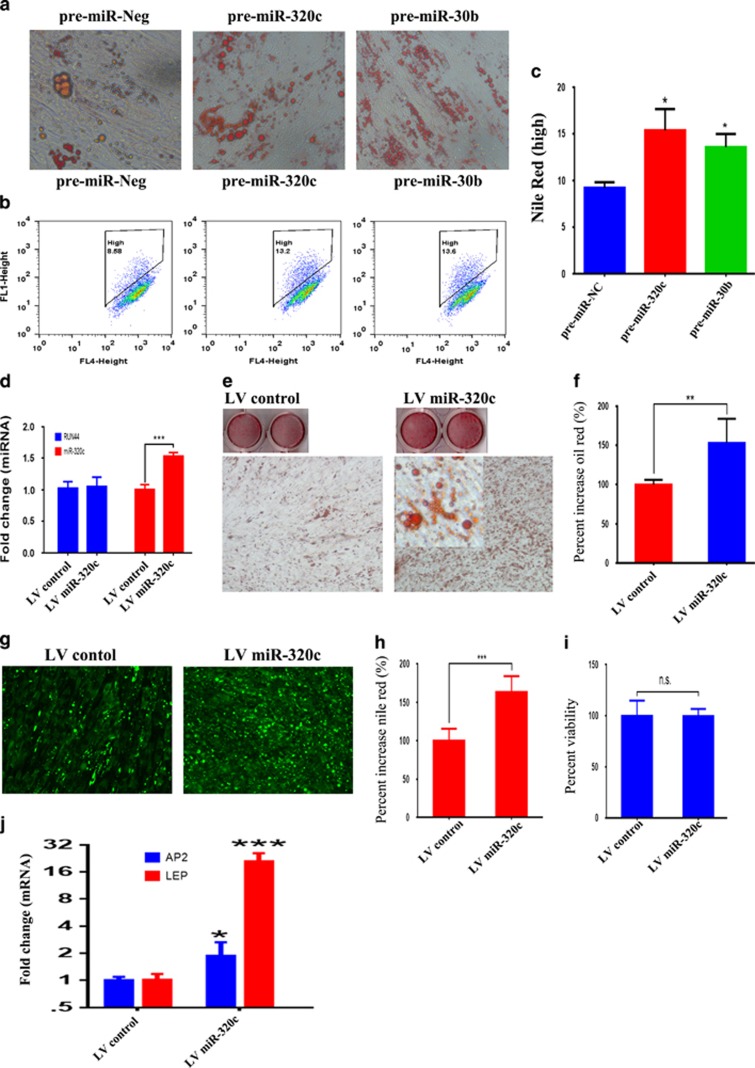
Forced expression of miR-320c- and miR-30b-enhanced AD differentiation of hMSCs. hMSCs were transfected with 30 nM of pre-miR-320c, pre-miR-30b and pre-miR-Neg, then were subjected to AD differentiation. (**a**) AD differentiation was assessed on day 7 using Oil Red O staining. (**b**) The percentage of Nile red^high^ cells was enumerated using flow cytometry. (**c**) Quantitative presentation of the data obtained in **b**. Data are presented as mean±S.E, *n*=3. (**d**) Stable expression of miR-320c in hMSC cells using lentiviral expression vector led to significant increase in miR-320c expression. (**e**) Oil Red O quantification in LV miR-320c and LV control cells after 7 days of adipocytic differentiation. (**g**) Nile red staining of LV miR-320c and LV control cells on day 7 adipocytic induction. The level of Nile red staining was quantified using molecular devices M5 microplate reader using fluorescence well-scan mode (**h**). Data are representative of three independent experiments, *n*=36. Cell numbers was quantified using the alamarBlue assay on LV miR-320c and LV control cells **(i)**, *n*=9**. (j)** Expression of adipo-specific markers in LV miR-320c or LV control cells after 7 days of adipocytic differentiation, *n*=6, *P<0.05, **P<0.005, ***P<0.0005

To examine for the potential role of selected miRNAs, miR-320c and -30b in regulating the adipocytic differentiation of hMSC, cells were transfected with pre-miR-320c, pre-miR-30b or pre-miR-negative control and subsequently were exposed to AIM. qRT-PCR revealed significant increase in miRNA expression in transfected cells (data nor shown). As shown in Figure 2a, cell transfected with pre-miR-320c and -30b exhibited enhanced formation of lipid-filled mature ADs. Concordant with those data, Nile red staining and fluorescence-activated cell scan (FACS) analysis revealed increased number of Nile Red High population in hMSC cultures transfected with pre-miR-320c and -30b compared with the controls (Figures 2b and c). As miR-320 family was the most novel family of miRNAs identified in current study as a possible regulator of adipocytic differentiation of hMSCs, all subsequent experiments focused on miR-320c member. In order to confirm that the enhanced adipocytic differentiation mediated via miR-320c was specific and not because of nonspecific effect as a result of transfection, we generated hMSCs stably expressing miR-320c using lentiviral-mediated transduction. As shown in Figures 2d and e, stable expression of miR-320c indeed led to enhanced adipocytic differentiation of hMSCs compared with cells transduced with control lentivirus. Representative images of Oil Red O staining are shown in Figure 2e, while quantification of Oil Red O staining demonstrated enhanced adipogenesis in LV miR-320c cells compared with control cells (Figure 2f). Similarly Nile red staining and quantification also demonstrated enhanced lipid droplet accumulation in miR-320c compared with control cells (Figures 2g and h). We observed no significant difference in cell viability on day 7 post-AD differentiation induction between LV miR-320c and LV control cells (Figure 2i), therefore the difference in Nile red staining is not due to difference in cells numbers. Concordant with that, the expression of AD-specific genes was higher in LV miR-320c cells compared with control cells (Figure 2j). Taken together, our data indicated enhanced adipocytic differentiation of hMSCs overexpressing miR-320c.

### Identification of bona fide mRNA targets for miR-320c

**Figure 3:**
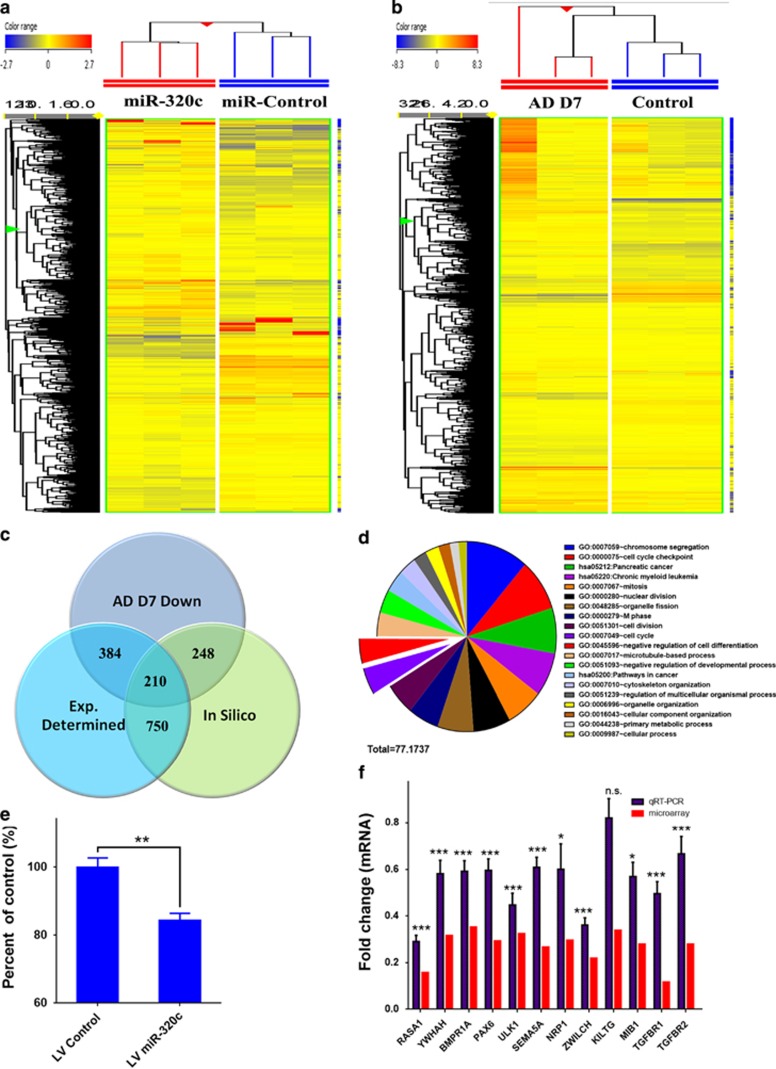
Identification of miR-320c bona fide gene targets during adipogenic differentiation of hMSCs. (**a**) Hierarchical clustering of hMSC transfected with miR-320c or control miRNA 72 h post transfection based on mRNA expression levels. Each column represents one replica. Expression level of each gene in a single sample is depicted according to the color scale. (**b**) Hierarchical clustering of control MSCs or MSCs differentiated into ADs (day 7) based on mRNA expression levels, where each column represents one replica. Expression level of each gene in a single sample is depicted according to the color scale. (**c**) Venn diagram demonstrating the overlap between experimentally determined miR-320c targets at baseline or following adipocytic differentiation of hMSC, and the *in silico*-predicted miR-320c targets based on TargetScan database. (**d**) Pie chart illustrating the distribution of the top 20 GO categories for the 210 predicted miR-320c gene targets. The pie section size corresponds to fold enrichment. (**e**) Quantification of cell viability of control hMSC or hMSC stably transduced with miR-320c LV on day 4 using alamarBlue assay. (**f**) qRT-PCR validation (blue) of selected miR-320c gene targets identified from microarray (red) (**c**). Data are presented as mean±S.E., *n*=6 from two experiments, *P<0.05, ***P<0.0005

In order to identify possible gene targets of miR-320c that regulate MSC differentiation into ADs, we used the following approach. hMSC transfected with miR-320c or control miRNA were cultured in presence or absence of AIM, total RNA was extracted from baseline and following 7 days of AD induction and subjected to microarray analysis. As shown in Figures 3a and b, hMSC overexpressing miR-320c did cluster together compared with cells transfected with miRNA control. Supplementary Tables 2 and 3 contain a list of genes that were downregulated (−1.3-fold, *P*<0.05) in hMSCs transfected with miR-320c compared with control cells at 72 h post transfection and in day 7 adipocytic induced hMSC, respectively. In addition to these genes that were experimentally determined, a third list of predicted miR-320c gene targets were curated from TargetScan database. The intersection in Venn diagram between three lists, identified 210 common genes, that is, genes that were downregulated upon miR-320c overexpression, downregulated during adipocytic differentiation of hMSCs and that were predicted to be targeted by miR-320c *in silico*. Gene ontology and pathway analysis of the identified 210 common genes showed strong enrichment for genes involved in regulating the cell cycle and cell differentiation (Figure 3d and Supplementary Table 4). Concordant with those data, we observed that un-induced hMSC stably expressing miR-320c had slower proliferation rate compared with control cells (Figure 3e). Several genes involved in cell cycle regulation and cell differentiation that were common to the three lists (MIB1, PAX6, ZWILCH, YWHAH and SEMA5A) or genes that were common to Exp. determined and *in silico* (TGFBR1, TGFBR2, NRP1, RASA1, ULK1, RUNX2 (Runt-related transcription factor 2), BMPR1A and KITLG) were chosen for further investigation. As confirmation of the microarray data, qRT-PCR showed good concordance between microarray and qRT-PCR except for one gene *KITLG* (Figure 3f).

### Validating the role of selected miR-320c targets in regulating adipocytic differentiation of hMSC

**Figure 4:**
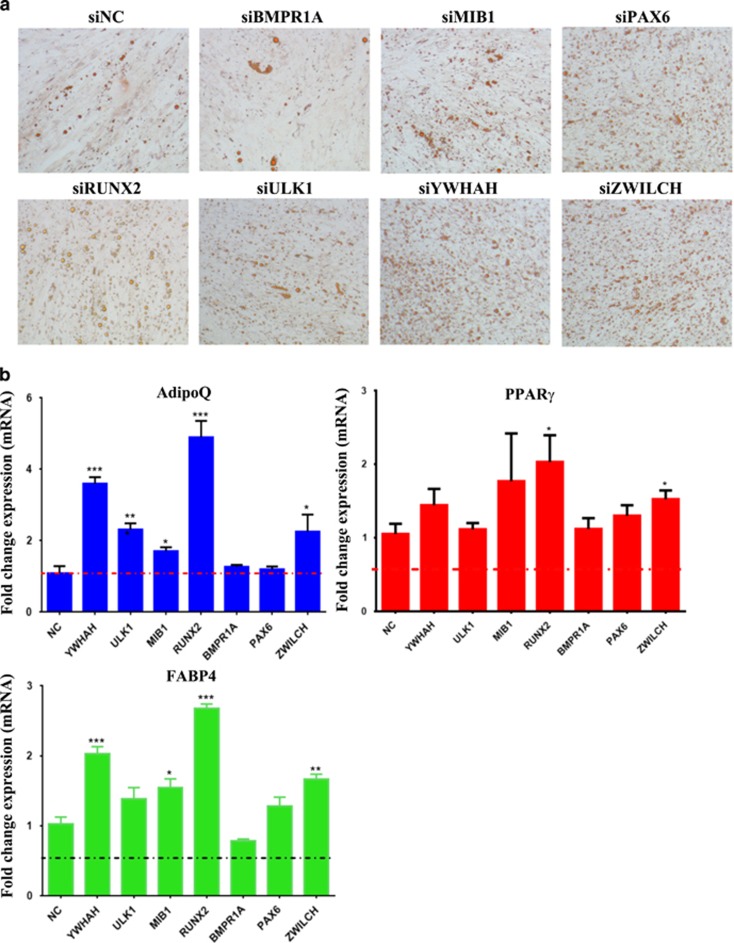
Functional validation of the identified miR-320c targets in regulating adipocytic differentiation of MSCs. hMSC were transfected with the indicated siRNA or control siRNA and were subjected to adipocytic differentiation induction for 7 days. (**a**) Oil Red O staining of mature lipid-filled ADs on day 7. (**b**) qRT-PCR analysis of AD marker genes (*AdipoQ*, *PPARγ* and *FABP4*). Gene expression was normalized to GAPDH and *β*-actin. Data are presented as mean fold change compared with cells transfected with control siRNA±S.E., *n*=6 from two independent experiments. *P<0. 05, **P<0.005, ***P<0.0005

To confirm whether the identified miR-320c targets are indeed involved in regulating adipocytic differentiation of hMSCs, we used siRNA approach to knock down the expression of selected genes. As shown in Figure 4a, siRNA-mediated silencing of MIB1, PAX6, RUNX2, YWHAH and ZWILCH led to increased number of adipocytic cells differentiated from hMSC. Concordant with that, qRT-PCR indicated upregulation of several adipocytic markers, AdipoQ, PPAR*γ* (peroxisome proliferator-activated receptor-*λ*) and FABP4, in cells transfected with YWHAH, MIB1, RUNX2 and ZWILCH siRNAs, suggesting a plausible role for these genes in miR-320-mediated effects on adipocytic differentiation of hMSC.

### miR-320c suppresses osteogenic differentiation of hMSCs

**Figure 5:**
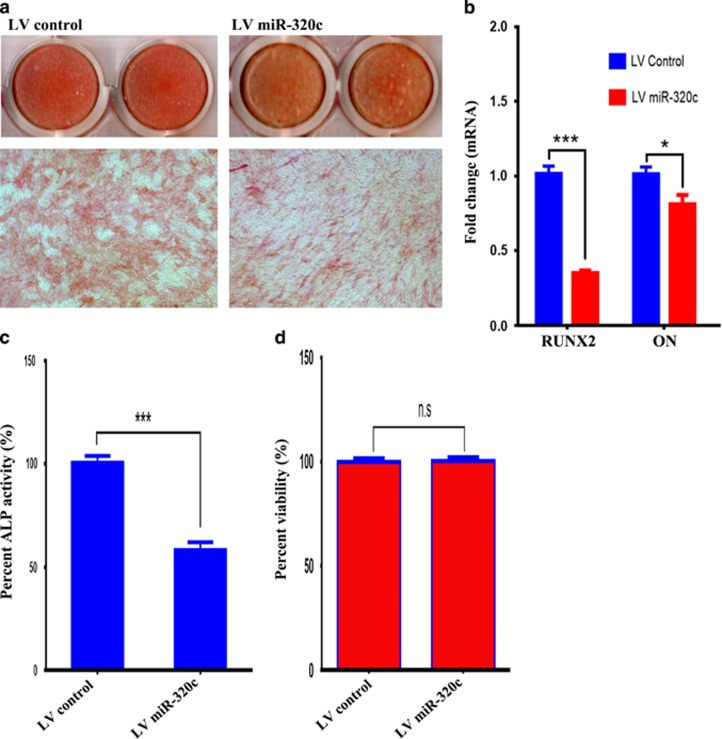
Overexpression of miR-320c suppressed ALP activity in hMSC. (**a**) Representative hMSC were stably transduced with a lentivirus containing miR-320c or control miRNA and were subjected to osteoblast differentiation induction for 10 days. ALP staining for control hMSC (LV control) or hMSC stably expressing miR-320c (LV miR-320c) is shown. (**b**) qRT-PCR analysis of osteoblast gene markers (RUNX2 and osteonectin). (**c**) Quantification of ALP activity on cells from **a**. Data is presented as relative ALP activity compared to cells transduced with LV control. Data are presented as mean±S.E. from five independent experiments, *n*=50 (**d**) Quantification of cell viability of LV control or LV miR-320c cells on day 10 post-osteogenic induction showing no significant difference between the two groups, *P<0.05, ***P<0.0005

Interestingly, RUNX2, which is a key transcription factor (TF) involved in osteogenesis, was among the novel gene targets identified for miR-320c family in current study. Therefore, we sought to assess the effect of miR-320c expression on osteogenic differentiation of hMSC. Data presented in Figure 5a showed lower ALP staining in LV miR-320c compared with LV control cell, as well as decreased expression of osteoblast marker genes, whereas the most reduction was seen for RUNX2 expression (Figure 5b). Similarly, ALP quantification revealed lower ALP activity on day 10 post-OB differentiation induction in LV miR-320c cells compared LV controls (Figure 5c). There was no difference in cell viability of OB-differentiated LV miR-320c and LV control hMSC (Figure 5d).

### Identification of RUNX2 as bone fide target for miR-320c during adipogenesis

**Figure 6:**
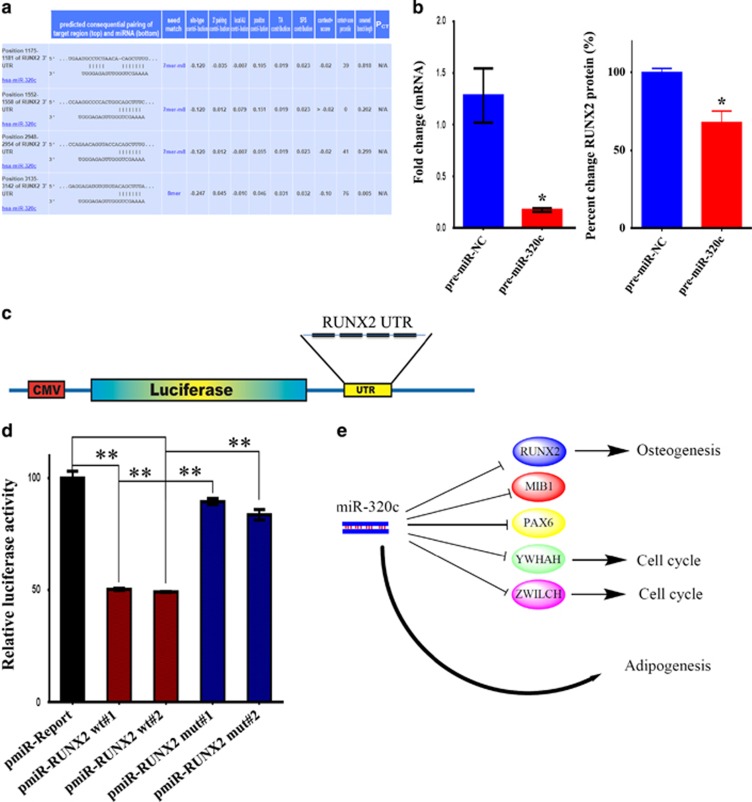
Direct regulation of RUNX2 by miR-320c. (**a**) Schematic presentation showing the alignment of miR-320c mature sequence and the putative binding sites within the 3′UTR region of the RUNX2 mRNA using TargetScan database. The exact positions of the interaction between RUNX2 3′UTR and miR-320c seed region are indicated. (**b**) Overexpression of miR-320c was associated with significant decrease in RUNX2 mRNA and protein. *P<0.05. (**c**) An illustration of the construction of luciferase reporter vector carrying the predicted RUNX2-miR-320c binding sites downstream of the firefly *luciferase* gene in the pMIR-REPORT vector. The number of predicted miR-320c binding sites within the 3′UTR region of RUNX2 is shown as black bars. (**d**) The indicated wild-type or mutant reporter vector was co-transfected with a pre-miR control (100 nM) or pre-miR-320c (100 nM) in HEK-293 cells, and luciferase activity was measured 24 h following transfection. Renilla luciferase activity was used for normalization. Data are presented as mean±S.E, *n*=6. **P<0.005. (**e**) A working model depicting the possible mechanisms by which miR-320c promotes adipocytic differentiation of hMSCs through targeting genes involved in multiple genetic pathways

Among the miR-320c-identified gene targets, RUNX2 was the most prominent. In addition to TargetScan, another database (HOCTAR, http://hoctar.tigem.it/), which examines for an inverse relationship between the expression of miRNA host gene and a potential target, was also used. We found that miR-320 family to be among the top 10% miRNAs predicted to regulate RUNX2, which further supports a role for miR-320 family in regulating RUNX2 expression during AD differentiation (Supplementary Table 5). Interestingly, RUNX2 had four predicted miR-320 family binding sites on it 3′-untranslated region (3′UTR) located between nucleotides 1175 and 3142 (Figure 6a). Overexpression of pre-miR-320c led to significant reduction in RUNX2 expression in hMSC (Figure 6b). To confirm that RUNX2 is indeed a direct target for miR-320 family, we constructed reporter vector carrying the predicted binding site(s) of RUNX2 downstream of a firefly *luciferase* gene in the pMIR-REPORTER miRNA Expression Reporter vector (Figure 6c).^24^ A mutant version of RUNX2 UTR reporter vector with mutations in the predicted miR-320 seed region(s) in the 3′UTRs was also generated using the primer combination listed in Table 2. The pRL-SV40 (encoding for renilla luciferase) was used for normalization. Co-transfection experiments in HEK-293 (human embryonic kidney 293) cells using two different RUNX2 UTR reporter clones demonstrated significant regulation of the RUNX2 reporter by miR-320c miRNA (~50% Figure 6d). The regulation of RUNX2 UTR by miR-320c was specific, as mutating the seed region completely abrogated this effect.

## Discussion

In the present study, we have identified miR-320 family as novel regulator of bone marrow-derived hMSC differentiation into ADs. Our data corroborate an increasing number of studies demonstrating the role of miRNAs in regulation of hMSC cell lineage fate as well as increasing number of other types of stem cells.^17,25^ Although several miRNA candidates that control osteoblastic differentiation of MSC have been described, only few miRNA have been reported to regulate their adipocytic differentiation. Among the reported miRNAs are miR-143, miR-138 and miR-637 that were implicated in regulating AD differentiation via modulation of ERK5, EID-1 and Osterix, respectively.^26, 27, 28^

In current study, we used an integrated analysis of miRNA expression profiling combined with bioinformatics analyses. Interestingly, several of the identified differentially regulated miRNAs during AD differentiation of hMSC have previously been reported to regulate hMSC differentiation (e.g., miR-222, miR-138 and miR-30 family^23,27,29,30^), indicating the importance of the regulatory network controlled by miRNAs as they are preserved across different cellular models of MSC.

We identified miR-320 family as the most prominent novel regulator of hMSC differentiation into ADs. Using Tri-Pronged approach combined with functional and biochemical assays, we identified several novel gene targets for miR-320 family during adipocytic differentiation of hMSC. Among these MIB1, PAX6, YWHAH, ZWILCH and RUNX2 were most relevant to adipogenesis. Interestingly, several of the identified genes are known to have a role in regulating cell proliferation and stem cell differentiation. For example, MIB1 and PAX6 were implicated in regulating neural stem cell differentiation.^31,32^ YWHAH has recently been implicated in regulating cell division during meiosis,^33^ while ZWILCH has been shown to be essential for kinetochore functions during cell division.^34^ However, our data revealed an additional role of these proteins in bone marrow adipogenesis. RUNX2 was one target identified in this study that is known for its being a master TF for inducing osteoblast differentiation. Therefore, it is plausible that miR-320 family promote adipogenesis *via* blocking other MSC differentiation pathways (i.e., osteoblast; Figure 6d).

Bioinformatics analysis revealed that RUNX2 3′ UTR harbors four potential binding sites for miR-320 family. Regulation of RUNX2 expression by miR-320 was subsequently confirmed using qRT-PCR and luciferase assay. The interaction between miR-320 and RUNX2 3′ UTR was found to be specific, as mutating miR-320 seed region in the 3′UTR of RUNX2 completely abrogated its regulatory effects. RUNX2 is osteoblast-specific TF that has an important role in MSC differentiation to osteoblasts.^35, 36, 37^ Previous studies have demonstrated that adipocytic differentiation of MSC is suppressed by RUNX2 and RUNX2^−/−^ calvarial cells exhibited an enhanced AD differentiation.^38^ Our data corroborate that osteoblast differentiation is the primary default differentiation pathway for bone marrow-derived hMSC and thus inhibition of RUNX2 activity is needed to promote AD differentiation. Interestingly, previous studies have also identified RUNX2 as a bona fide target for pro-adipocytic miRNAs such as miR-30a, 30d and miR-204/211. Therefore, RUNX2 appears to be a key negative regulator of adipogenesis that seems to be targeted by several miRNA families, including the miR-320 family in our study. This is not surprising as RUNX2 has relatively large 3′UTR (3.777 kb) that makes it a likely target for several groups of miRNAs. Interestingly, lentiviral-medicated stable expression of miR-320c at physiological levels (~1.5-fold; Figure 2d) also promoted adipocytic differentiation of hMSC, thus further supporting that the observed effects have physiological relevance.

MSC commitment to specific lineage, AD or osteoblast, was shown to be regulated by the expression of different TFs, which are involved in different cellular pathways. MSCs express several adipogenic TFs, for example, CCAAT-enhancer-binding protein (C/EBP) and PPAR*γ*, as well as osteoblastic TF, for example, RUNX2, MSX2, DLX5 and Osterix.^39, 40, 41, 42, 43, 44, 45, 46^ It is plausible that the undifferentiated state of MSC is maintained by suppression of lineage-specific TFs. Most of the studies focused on the ability of miRNA to induce lineage-specific differentiation through induction of lineage-specific TFs. Our study suggests that suppressing of the TFs belonging to an alternative differentiation lineage is an important mechanism controlling MSC lineage fate choice. miRNA can thus be a target for pharmacological intervention to control lineage fate of MSC.

## Materials and Methods

### Cell culture

As a model for primary human bone marrow-derived MSC (hMSC), we used a Telomerized hMSC line that has been created through overexpression of human telomerase reverse transcriptase gene (hTERT) transduction (hMSC-TERT).^47^ The hMSC-TERT expresses all known markers of primary hMSCs^48,49^ and exhibit ‘stemmness' characteristics by being able to form bone and bone marrow microenvironment when implanted *in vivo*.^19^ The hMSC-TERT cells were cultured in Dulbecco's modified Eagle's medium (DMEM) supplemented with D-glucose 4500 mg/l, 4 mM L-glutamine and 110 mg/l sodium pyruvate, 10% fetal bovine serum (FBS), 1% penicillin–streptomycin (Pen–Strep) and non-essential amino acids 1. For transfection studies, HEK-293 cells were used and the cells were cultured in the same culture medium as above. All reagents were provided from Gibco–Invitrogen (Carlsbad, CA, USA). All cells were incubated in a humidified atmosphere containing 95% air and 5% CO_2_ at 37 °C, medium was replaced once a week or as needed.

### Adipogenic differentiation of hMSCs

hMSC-TERT cells were cultured in basal medium in 24-well tissue culture plates. When cells reached 80–90% confluence, medium was replaced with AIM (DMEM medium supplemented with 10% FBS, 10% horse serum (Sigma, St Louis, MO, USA), 1% Pen–Strep, 100 nM dexamethasone, 0.45 mM isobutyl methyl xanthine (Sigma), 3 *μ*g/ml insulin (Sigma) and 1 *μ*M Rosiglitazone (BRL49653). The AIM was replaced every 3 days. Control cells were cultured in parallel in normal DMEM medium. Cells were assessed for adipogenic differentiation on days 7 and 13 post differentiation.

### Osteogenic differentiation of hMSCs

For osteogenic differentiation of hMSCs, cells were cultured as above then were exposed to osteogenic induction medium (DMEM containing 10% FBS, 1% Pen–Strep,50 *μ*g/ml L-ascorbic acid (Wako Chemicals GmbH, Neuss, Germany), 10 mM *β*-glycerophosphate (Sigma) and 10 nM calcitriol (1*α*,25-dihydroxy vitamin D3; Sigma) and 10 nM Dexamethasone (Sigma)).

### Lentiviral transduction

Lentiviral particles encoding for has-miR-320c-1 (LP-HmiR0470-MR03-0200-S) or control lentiviral particles (LP-MCHR-LV105-0200) were purchased from Genecopoeia (Genecopoeia Inc., Rockville, MD, USA). Hundred thousand hMSCs were seeded in complete DMEM in 24-well plate. Forty-eight hours later (~80 confluency), media was removed and then 20 *μ*l of crude lentiviral particles in 500 *μ*l of DMEM+5% heat-inactivated serum (Invitrogen) and 1% Pen–Strep supplemented with polybrene (8 *μ*g/ml; Sigma) was added to the cells. Seventy-two hours later, media was removed and transduced cells were selected with puromycin (1 *μ*g/ml, Sigma) for 1 week until stably transduced cells were generated.

### Total RNA isolation and quantification of miRNA and mRNA expression

Total RNA containing the small RNA fraction were isolated from hMSC-TERT cells using Total RNA Purification Kit (Norgen-Biotek Corp., Thorold, ON, Canada) according to the manufacturer's instructions. The concentrations of total RNA were measured using NanoDrop 2000 (Thermo Scientific, Wilmington, DE, USA). qRT-PCR analysis was performed as previously described^50^ to assess the expression levels of miRNAs using TaqMan miRNA Assays (Applied Biosystems Inc., Foster City, CA, USA). In brief, 10 ng of total RNA was subjected to reverse transcription using miRNA-specific primers supplied by ABI. Subsequently, second set of miRNA-specific primers was used to amplify each miRNA according to the manufacturer's recommendations. Expression levels of adipogenic-related genes, *PPARγ,* *LEP*, *AP2* and *AdipoQ*, were assessed using qRT-PCR. Reverse transcription was performed on 500 ng of total RNA using High Capacity Reverse Transcriptase Kit (Applied Biosystems Inc.) according to manufacturer's specifications. qRT-PCR was done using FAST-SYBR Green Master Mix (Applied Biosystems Inc.) and the StepOne Plus Real-Time PCR Detection System (Applied Biosystems Inc.). Primers used for gene expression analysis are listed in Table 1 and were either previously published or were designed using NCBI Primer-BLAST (http://www.ncbi.nlm.nih.gov/tools/primer-blast/). The 2▵CT value method was used to calculate relative expression of miRNAs and mRNAs.^51^

### miRNA expression profiling

All miRNA microarray experiments and analyses were conducted by Exiqon (Copenhagen, Denmark). hMSC-TERT cells were differentiated into ADs, and on days 0, 7 and 13 RNA was extracted as described above.^19^ The samples were labeled using the miRCURY LNA microRNA Hi-Power Labelling Kit, Hy3/Hy5, and hybridized on the miRCURY LNA microRNA Array (6th GEN) as outlined in Supplementary Figure 1. Samples that did not meet quality requirements were excluded from the data analyses. Data were normalized using the global Lowess regression algorithm, then were subjected to unsupervised as well as supervised data analysis. *P*-values were corrected for multiple testing using the Bonferroni adjustment method. Subsequently, miRNAs found to be significantly regulated by the one-way ANOVA test were subjected to the Tukey's ‘Honest Significant Difference' test to determine which groups contribute most to the significant difference. All data analyses were done using R/bioconductor software. Gene ontology analyses were conducted using DAVID Bioinformatics Database functional-annotation tools, as we have previously described.^52^ miRNA expression data sets were deposited to the Gene Expression Omnibus (GEO), accession number GSE59684.

### miRNA and siRNA transfection experiments

To investigate the role of selected miRNAs in regulating hMSC differentiation into ADs, hMSC cells were transfected with the indicated miRNA precursors (pre-miR-Neg, pre-miR-320c and pre-miR30b, Ambion, Foster City, CA, USA) or siRNAs (Ambion) using reverse transfection protocol and Lipofectamine 2000 (Invitrogen) as we previously described.^52^ In brief, 0.05 × 10^6^ cells were reverse-transfected with 30 nM of the indicated pre-miRs or siRNAs complexed with 1.5 *μ*l of Lipofectamine 2000 in a 24-well tissue culture plate. Transfection cocktail was subsequently replaced after 4 h with normal DMEM without antibiotics. On day 3, medium was replaced with DMEM–AIM as described above and fresh induction medium was replaced every 3 days.

### Cloning of RUNX2 3′UTR and luciferase assay

A reporter vector (pMir-Report, ABI) carrying the predicted miR-320 binding site(s) from RUNX2 3′UTR was constructed using partially complementary primer pairs listed in Table 1. Amplification was conducted as we previously described^24^ and using Amplitaq gold DNA polymerase (Applied Biosystems Inc.). As positive control, we constructed a vector carrying the full-length complementary sequence to Let-7b miRNA. A mutant version of RUNX2 3′UTR reporter plasmid was generated by mutating the seed region for the miR-320 miRNA family using the indicated primers in Table 1. All regions were subsequently cloned into the *Spe*I and *Hind*III sites downstream of the firefly *luciferase* gene in the pMIR-REPORT vector (Applied Biosystems Inc.). To assess the direct interaction between miR-320 miRNA family and the 3′UTR from RUNX2, HEK-293 cells were co-transfected with 100 nM of pre-miR-Neg or pre-miR-320c and 100 ng of pMIR-REPORT carrying either wt or mutant 3′UTR sequences, along with 20 ng of pRL-SV40 vector (Promega, Madison, WI, USA) carrying the *Renilla luciferase* gene. Transfection experiments were conducted using Lipofectamine 2000 (Invitrogen). At 48 h post transfection, luciferase activity was measured using the Dual-Glo luciferase assay system (Promega). Firefly luciferase activity was then normalized to that of *Renilla* luciferase.

### Gene expression microarray

hMSC were differentiated into ADs as described above. On day 7, total RNA was extracted using Total RNA Purification Kit (Norgen-Biotek Corp.) according to the manufacturer's instructions. For miR-320c-transfected cells, MSCs were transfected with either pre-miR-control or pre-miR-320c. Seventy-two hours later, total RNA was isolated as described above. The concentrations of total RNA were measured using NanoDrop 2000 (Thermo Scientific). Extracted RNA was labeled and then hybridized to the Agilent Human SurePrint G3 Human GE 8 × 60 k microarray chip (Agilent Technologies, Santa Clara, CA, USA). All microarray experiments were conducted at the Microarray Core Facility (Stem Cell Unit, King Saud University College of Medicine, Riyadh, Saudi Arabia). Data analyses were conducted using GeneSpring 12.0 software (Agilent Technologies) and DAVID bioinformatic tool as described before.^52,53^ Percentile Shift was used for data normalization while Benjamini–Hochberg false discovery rate method was used for multiple testing corrections. The gene expression profiling in hMSCs transfected with miR-320c and the gene expression profiling during adipogenic differentiation of hMSCs data sets were deposited to the GEO, accession numbers GSE59458 and GSE59450, respectively.

### Oil Red O staining for ADs

At the indicated time points, adipogenic differentiation was determined by Oil Red O staining for lipid-filled mature ADs. Cells were washed with phosphate-buffered saline (PBS), fixed with 4% paraformaldehyde for 10 min and were incubated with a newly made and filtered (0.45 *μ*M) Oil Red O staining solution (Sigma; 0.05 g in 60% isopropanol) for 1 h at room temperature. Photomicrographs were acquired using inverted Zeiss microscope (Thornwood, NY, USA).

### AD enumeration by flow cytometry

Nile Red Staining was performed as we described previously.^54^ In brief, following trypsinization, the cells were washed with calcium and magnesium-free PBS. Subsequently, Nile Red dye (N3013; Sigma) was added at a final concentration of l00 ng/ml. Following 5 min incubation at 4 °C, the cells were washed in PBS, centrifuged and re-suspended in 500 *μ*l PBS and were analyzed using BD FACSCalibur flow cytometer (BD Biosciences, Franklin Lakes, NJ, USA). Staining was detected in the green fluorescence channel (FL1); the gating strategy is presented in Figure 2. Data were analyzed using FlowJo software (Tree Star, Ashland, OR, USA).

### Nile red fluorescence determination and quantification of adipogenesis using microplate reader

Stock solution of Nile red (1 mg/ml) in DMSO was prepared and stored at −20 °C protected from light. Staining was performed on unfixed cells. Cultured undifferentiated and differentiated cells (were grown in Corning polystyrene flat bottom 96-well TC-treated black microplates, Corning, NY, USA) were washed once with PBS. The dye was then added directly to the cells (5 *μ*g/ml in PBS), and the preparation was incubated for 10 min at room temperature then washed twice with PBS. Fluorescent signal was measured using SpectraMax/M5 fluorescence spectrophotometer plate reader (Molecular Devices Co., Sunnyvale, CA, USA) using bottom well-scan mode where nine readings were taken per well using Ex (485 nm) and Em (572 nm) spectra. Furthermore, fluorescence images were taken using FLoid cell imaging station (Life Technologies Inc., Grand Island, CA, USA).

### ALP activity quantification

To quantify ALP activity in control and differentiated hMSC, we used the BioVision ALP activity colorimetric assay kit (BioVision, Inc., Milpitas, CA, USA) with some modifications. Cells were cultured in 96-well plates under normal or osteogenic induction conditions, then on day 10, wells were rinsed once with PBS and were fixed using 3.7% formaldehyde in 90% ethanol for 30 s at room temperature. Subsequently, fixative was removed and 50 *μ*l of pNPP solution was added to each well and incubated for 1 h in the dark at room temperature. Reaction was subsequently stopped by adding 20 *μ*l stop solution and gently shaking the plate. O.D. was then measured at 405 nm using SpectraMax/M5 fluorescence spectrophotometer plate reader.

### RUNX2 quantification

For quantification of RUNX2 protein, hMSC were transfected with pre-miR-Neg or pre-miR-320c (30 nM), and 72 h later cells were collected and washed with PBS. Cells were lysed in 100 *μ*l PBS containing protease inhibitors using five freeze-thaw cycles. Cell lysate was subsequently spun down at maximum speed for 10 min, and supernatant was collected and stored at −80°C. Subsequently, RUNX2 was quantified using the RUNX2 ELISA kit according to the manufacturer's recommendation (Uscn Life Science Inc., Wuhan, PRC).

### AlamarBlue cell viability assay

Cell viability was measured using alamarBlue assay according to the manufacturer's recommendations (AbD Serotec, Raleigh, NC, USA). In brief, we cultured cells in 96-well plates in 100 *μ*l of the appropriate medium and at the indicated time point, and 10 *μ*l of alamarBlue substrate was added and plates were incubated in the dark at 37 °C for 1h. Reading was subsequently taken using fluorescent mode (Ex 530 nm/Em 590 nm) using BioTek Synergy II microplate reader (BioTek Inc., Winooski, VT, USA).

### Statistics

Statistical analyses and graphing were performed using Microsoft excel 2010 and GraphPad Prism 6.0 software (Graphpad software, San Diego, CA, USA). *P*-values were calculated using the two-tailed *t*-test.

## Figures and Tables

**Table 1:** Significantly differentially expressed miRNAs on AD day 13 *versus* AD day 0

*No.*	*Annotation*	*logFC*	*AvgExpr*	*AvgHy3*	*P-value*	*Adj. P-value*
1	hsa-miR-320c	1.200823	0.234397	8.424051	6.39E−07	0.00026
2	hsa-miR-30b	1.615031	0.310867	8.365077	3.12E−06	0.0013
3	hsa-miR-320e	1.206532	0.222737	8.432005	5.03E−06	0.0021
4	hsa-miR-320a	1.182796	0.204731	8.699699	5.64E−06	0.0023
5	hsa-miR-320b	1.248647	0.139694	8.734353	8.83E−06	0.0037
6	hsa-miR-29c	2.620266	0.310883	8.044084	1.32E−05	0.0055
7	hsa-miR-10b	1.383596	0.156777	8.122331	1.37E−05	0.0057
8	hsa-miR-19a	0.646275	0.285073	8.533174	1.47E−05	0.0061
9	hsa-miR-101	2.644133	-0.05242	8.501706	1.63E−05	0.0068
10	hsa-miR-320d	1.178505	0.28873	8.23224	1.71E−05	0.0072
11	hsa-miR-148b	0.803186	0.294123	6.764786	2.19E−05	0.0092
12	hsa-miR-29a	1.228473	0.347	12.13131	2.29E−05	0.0096
13	hsa-miR-193b	1.192514	0.18976	8.324068	2.44E−05	0.010
14	hsa-miR-151-5p	0.701698	0.079185	7.519235	2.51E−05	0.010
15	hsa-miR-129*	1.434831	0.064513	6.293654	3.11E−05	0.013
16	hsa-miR-193b*	0.392681	0.04686	5.884442	3.59E−05	0.015
17	hsa-miR-30c	1.44055	0.202695	8.307646	3.74E−05	0.015
18	hsa-miR-374b	1.202467	0.155924	7.191527	3.76E−05	0.015
19	hsa-miR-365	1.24017	0.331153	8.881532	4.11E−05	0.017
20	hsa-miR-103a	0.989911	0.22999	8.47661	4.64E−05	0.019
21	hsa-miR-3647-5p	0.715019	−0.17482	6.152502	4.66E−05	0.019
22	hsa-miR-30e	1.047744	0.480218	7.490472	5.47E−05	0.023
23	hsa-miR-3647-3p	0.872259	0.087001	8.168902	5.85E−05	0.024
24	hsa-miR-190	1.532023	0.435949	6.418949	5.96E−05	0.025
25	hsa-miR-222	−1.15414	-0.14702	10.71594	6.03E−05	0.025
26	hsa-miR-152	0.717162	0.380272	7.531555	6.32E−05	0.026
27	hsa-miR-374a	1.285674	0.256238	7.48288	6.46E−05	0.027
28	hsa-miR-26b	1.519303	0.156895	8.870564	6.49E−05	0.027
29	hsa-miR-28-5p	0.686888	0.012992	5.912515	6.82E−05	0.028
30	hsa-miR-186	1.166416	0.40042	6.430794	7.04E−05	0.029
31	hsa-miR-191	1.222257	0.357161	7.655313	7.42E−05	0.031
32	hsa-miR-26a	1.611705	0.16245	7.403967	7.54E−05	0.031
33	hsa-miR-30a	1.400624	0.282938	7.650121	8.02E−05	0.033
34	hsa-miR-16	0.831693	0.543501	10.61485	8.99E−05	0.037
35	hsa-miR-15a	0.969761	0.440476	9.501428	9.15E−05	0.038
36	hsa-miR-1285	−1.24066	−0.31694	7.180472	9.24E−05	0.038
37	hsa-miR-151-3p	0.491623	0.195363	6.432898	9.56E−05	0.040
38	hsa-miR-30d	1.343111	0.511484	7.202426	0.000103	0.043

**Table 2:** Primer Sequences used for cloning and qRT-PCR

*No.*	*Name*		*Sequence*
*A Cloning primers*			
1	RUNX2 wt UTR	F	5′GTTGTTACTAGTTCTTTGAATGCCTCTAACACAGCTTTGCCTTTACCCAAGGCC CCACTGGCAGCTTTCCACATATCAGAGTTCCAGA
		R	5′GTTGTTAAGCTTTCCTTAAAGCTGTACACACATCTCCTCAAACCAAAGCTGTGGTACCTGTTCTGGAACTCTGATATGTGGAAAGCTG
2	RUNX2 mut UTR	F	5′GTTGTTACTAGTTCTTTGAATGCCTCTAACAa**gaaggg**GCCTTTACCCAAGGCCCCACTGGa**gaaggg**CCACATATCAGAGTTCCAGA
		R	5′GTTGTTAAGCTTTCCTT**cccttct**TACACACATCTCCTCAAACC**cccttct**TGGTACCTGTTCTGGAACTCTGATATGTGG**cccttct**
			
*B qRT-PCR primers*			
1	AP2	F	5′ TGGTTGATTTTCCATCCCAT
		R	5′ GCCAGGAATTTGACGAAGTC
2	PPARγ	F	5′ GCTTCTGGATTTCACTATGG
		R	5′ AAACCTGATGGCATTATGAG
3	AdipoQ	F	5′ GCAGTCTGTGGTTCTGATTCCATAC
		R	5′ GCCCTTGAGTCGTGGTTTCC
4	LEP	F	5′ CAGCGGTTGCAAGGCCCAAGA
		R	5′ GGCCAAAGCCACAAGAATCCGC
5	GAPDH	F	5′ CTGGTAAAGTGGATATTGTTGCCAT
		R	5′ TGGAATCATATTGGAACATGTAAACC
6	ALPL	F	5′ GACGGACCCTCGCCAGTGCT
		R	5′ AATCGACGTGGGTGGGAGGGG
7	Osteonectin	F	5′ GAGGAAACCGAAGAGGAGG
		R	5′ GGGGTGTTGTTCTCATCCAG

Lower case letter indicate sites of mutations in seed regions

## Abbreviations

3'UTR: 3'-Untranslated region
C/EBP: CCAAT-enhancer-binding protein
DMEM: Dulbecco's modified Eagle's medium
FACS: Fluorescence-activated cell scan
FBS: Fetal bovine serum
HEK-293: Human embryonic kidney 293 cells
hMSCs: Human mesenchymal stem cells
hTERT: Human telomerase reverse transcriptase
miRNAs: MicroRNAs
MSCs: Mesenchymal stem cells
PBS: Phosphate-buffered saline
PPAR*γ*: Peroxisome proliferator-activated receptor-*γ*
qRT PCR: Quantitative real-time reverse transcription PCR
RUNX2: Runt-related transcription factor 2
MIB1: Mindbomb E3 ubiquitin protein ligase 1
PAX6: Paired box 6

## References

[bib1] 1Naveiras O, Nardi V, Wenzel PL, Hauschka PV, Fahey F, Daley GQ. Bone-marrow adipocytes as negative regulators of the haematopoietic microenvironment. Nature 2009; 460: 259–263. 19516257 10.1038/nature08099 PMC2831539

[bib2] 2Gimble JM, Robinson CE, Wu X, Kelly KA. The function of adipocytes in the bone marrow stroma: an update. Bone 1996; 19: 421–428. 8922639 10.1016/s8756-3282(96)00258-x

[bib3] 3Menagh PJ, Turner RT, Jump DB, Wong CP, Lowry MB, Yakar S et al. Growth hormone regulates the balance between bone formation and bone marrow adiposity. J Bone Miner Res 2010; 25: 757–768. 19821771 10.1359/jbmr.091015 PMC3153330

[bib4] 4Syed FA, Oursler MJ, Hefferanm TE, Peterson JM, Riggs BL, Khosla S. Effects of estrogen therapy on bone marrow adipocytes in postmenopausal osteoporotic women. Osteoporos Int 2008; 19: 1323–1330. 18274695 10.1007/s00198-008-0574-6 PMC2652842

[bib5] 5Justesen J, Stenderup K, Ebbesen EN, Mosekilde L, Steiniche T, Kassem M. Adipocyte tissue volume in bone marrow is increased with aging and in patients with osteoporosis. Biogerontology 2001; 2: 165–171. 11708718 10.1023/a:1011513223894

[bib6] 6Fazeli PK, Horowitz MC, MacDougald OA, Scheller EL, Rodeheffer MS, Rosen CJ et al. Marrow fat and bone—new perspectives. J Clin Endocrinol Metab 2013; 98: 935–945. 23393168 10.1210/jc.2012-3634 PMC3590487

[bib7] 7Aldahmash A, Zaher W, Al-Nbaheen M, Kassem M. Human stromal (mesenchymal) stem cells: basic biology and current clinical use for tissue regeneration. Ann Saudi Med 2012; 32: 68–77. 22156642 10.5144/0256-4947.2012.68 PMC6087654

[bib8] 8Gimble JM, Zvonic S, Floyd ZE, Kassem M, Nuttall ME. Playing with bone and fat. J Cell Biochem 2006; 98: 251–266. 16479589 10.1002/jcb.20777

[bib9] 9Perera RJ, Ray A. MicroRNAs in the search for understanding human diseases. BioDrugs 2007; 21: 97–104. 17402793 10.2165/00063030-200721020-00004

[bib10] 10Lakshmipathy U, Hart RP. Concise review: microRNA expression in multipotent mesenchymal stromal cells. Stem Cells 2008; 26: 356–363. 17991914 10.1634/stemcells.2007-0625 PMC2673465

[bib11] 11Krichevsky AM, Sonntag KC, Isacson O, Kosik KS. Specific microRNAs modulate embryonic stem cell-derived neurogenesis. Stem Cells 2006; 24: 857–864. 16357340 10.1634/stemcells.2005-0441 PMC2605651

[bib12] 12Chen JF, Mandel EM, Thomson JM, Wu Q, Callis TE, Hammond SM et al. The role of microRNA-1 and microRNA-133 in skeletal muscle proliferation and differentiation. Nat Genet 2006; 38: 228–233. 16380711 10.1038/ng1725 PMC2538576

[bib13] 13Zhao Y, Samal E, Srivastava D. Serum response factor regulates a muscle-specific microRNA that targets Hand2 during cardiogenesis. Nature 2005; 436: 214–220. 15951802 10.1038/nature03817

[bib14] 14Pedersen I, David M. MicroRNAs in the immune response. Cytokine 2008; 43: 391–394. 18701320 10.1016/j.cyto.2008.07.016 PMC3642994

[bib15] 15Alajez NM. Cancer stem cells. From characterization to therapeutic implications. Saudi Med J 2011; 32: 1229–1234. 22159375

[bib16] 16Kloosterman WP, Lagendijk AK, Ketting RF, Moulton JD, Plasterk RH. Targeted inhibition of miRNA maturation with morpholinos reveals a role for miR-375 in pancreatic islet development. PLoS Biol 2007; 5: e203. 17676975 10.1371/journal.pbio.0050203 PMC1925136

[bib17] 17Tay YM, Tam WL, Ang YS, Gaughwin PM, Yang H, Wang W et al. MicroRNA-134 modulates the differentiation of mouse embryonic stem cells, where it causes post-transcriptional attenuation of Nanog and LRH1. Stem Cells 2008; 26: 17–29. 17916804 10.1634/stemcells.2007-0295

[bib18] 18Taipaleenmaki H, Bjerre Hokland L, Chen L, Kauppinen S, Kassem M. Mechanisms in endocrinology: micro-RNAs: targets for enhancing osteoblast differentiation and bone formation. Eur J Endocrinol 2012; 166: 359–371. 22084154 10.1530/EJE-11-0646

[bib19] 19Eskildsen T, Taipaleenmaki H, Stenvang J, Abdallah BM, Ditzel N, Nossent AY et al. MicroRNA-138 regulates osteogenic differentiation of human stromal (mesenchymal) stem cells *in vivo*. Proc Natl Acad Sci USA 2011; 108: 6139–6144. 21444814 10.1073/pnas.1016758108 PMC3076836

[bib20] 20Zeng Y, Qu X, Li H, Huang S, Wang S, Xu Q et al. MicroRNA-100 regulates osteogenic differentiation of human adipose-derived mesenchymal stem cells by targeting BMPR2. FEBS Lett 2012; 586: 2375–2381. 22684006 10.1016/j.febslet.2012.05.049

[bib21] 21Tuddenham L, Wheeler G, Ntounia-Fousara S, Waters J, Hajihosseini MK, Clark I et al. The cartilage specific microRNA-140 targets histone deacetylase 4 in mouse cells. FEBS Lett 2006; 580: 4214–4217. 16828749 10.1016/j.febslet.2006.06.080

[bib22] 22Laine SK, Alm JJ, Virtanen SP, Aro HT, Laitala-Leinonen TK. MicroRNAs miR-96, miR-124, and miR-199a regulate gene expression in human bone marrow-derived mesenchymal stem cells. J Cell Biochem 2012; 113: 2687–2695. 22441842 10.1002/jcb.24144

[bib23] 23Skarn M, Namlos HM, Noordhuis P, Wang MY, Meza-Zepeda LA, Myklebost O. Adipocyte differentiation of human bone marrow-derived stromal cells is modulated by microRNA-155, microRNA-221, and microRNA-222. Stem Cells Dev 2012; 21: 873–883. 21756067 10.1089/scd.2010.0503

[bib24] 24Alajez NM, Shi W, Wong D, Lenarduzzi M, Waldron J, Weinreb I et al. Lin28b promotes head and neck cancer progression via modulation of the insulin-like growth factor survival pathway. Oncotarget 2012; 3: 1641–1652. 23482325 10.18632/oncotarget.785 PMC3681501

[bib25] 25Tome M, Lopez-Romero P, Albo C, Sepulveda JC, Fernandez-Gutierrez B, Dopazo A et al. miR-335 orchestrates cell proliferation, migration and differentiation in human mesenchymal stem cells. Cell Death Differ 2011; 18: 985–995. 21164520 10.1038/cdd.2010.167 PMC3131940

[bib26] 26Esau C, Kang X, Peralta E, Hanson E, Marcusson EG, Ravichandran LV et al. MicroRNA-143 regulates adipocyte differentiation. J Biol Chem 2004; 279: 52361–52365. 15504739 10.1074/jbc.C400438200

[bib27] 27Yang Z, Bian C, Zhou H, Huang S, Wang S, Liao L et al. MicroRNA hsa-miR-138 inhibits adipogenic differentiation of human adipose tissue-derived mesenchymal stem cells through adenovirus EID-1. Stem Cells Dev 2011; 20: 259–267. 20486779 10.1089/scd.2010.0072

[bib28] 28Zhang JF, Fu WM, He ML, Wang H, Wang WM, Yu SC et al. MiR-637 maintains the balance between adipocytes and osteoblasts by directly targeting Osterix. Mol Biol Cell 2011; 22: 3955–3961. 21880893 10.1091/mbc.E11-04-0356 PMC3204058

[bib29] 29Zaragosi LE, Wdziekonski B, Brigand KL, Villageois P, Mari B, Waldmann R et al. Small RNA sequencing reveals miR-642a-3p as a novel adipocyte-specific microRNA and miR-30 as a key regulator of human adipogenesis. Genome Biol 2011; 12: R64. 21767385 10.1186/gb-2011-12-7-r64 PMC3218826

[bib30] 30Karbiener M, Neuhold C, Opriessnig P, Prokesch A, Bogner-Strauss JG, Scheideler M. MicroRNA-30c promotes human adipocyte differentiation and co-represses PAI-1 and ALK2. RNA Biol 2011; 8: 850–860. 21878751 10.4161/rna.8.5.16153

[bib31] 31Barsi JC, Rajendra R, Wu JI, Artzt K. Mind bomb1 is a ubiquitin ligase essential for mouse embryonic development and Notch signaling. Mech Dev 2005; 122: 1106–1117. 16061358 10.1016/j.mod.2005.06.005

[bib32] 32St-Onge L, Sosa-Pineda B, Chowdhury K, Mansouri A, Gruss P. Pax6 is required for differentiation of glucagon-producing alpha-cells in mouse pancreas. Nature 1997; 387: 406–409. 9163426 10.1038/387406a0

[bib33] 33De S, Kline D. Evidence for the requirement of 14-3-3eta (YWHAH) in meiotic spindle assembly during mouse oocyte maturation. BMC Dev Biol 2013; 13: 10. 23547714 10.1186/1471-213X-13-10 PMC3620909

[bib34] 34Williams BC, Li Z, Liu S, Williams EV, Leung G, Yen TJ et al. Zwilch, a new component of the ZW10/ROD complex required for kinetochore functions. Mol Biol Cell 2003; 14: 1379–1391. 12686595 10.1091/mbc.E02-09-0624 PMC153108

[bib35] 35Bialek P, Kern B, Yang X, Schrock M, Sosic D, Hong N et al. A twist code determines the onset of osteoblast differentiation. Dev Cell 2004; 6: 423–435. 15030764 10.1016/s1534-5807(04)00058-9

[bib36] 36Shen R, Wang X, Drissi H, Liu F, O'Keefe RJ, Chen D. Cyclin D1-cdk4 induce runx2 ubiquitination and degradation. J Biol Chem 2006; 281: 16347–16353. 16613857 10.1074/jbc.M603439200 PMC2649830

[bib37] 37Muruganandan S, Roman AA, Sinal CJ. Adipocyte differentiation of bone marrow-derived mesenchymal stem cells: cross talk with the osteoblastogenic program. Cell Mol Life Sci 2009; 66: 236–253. 18854943 10.1007/s00018-008-8429-z PMC11131547

[bib38] 38Kobayashi H, Gao Y, Ueta C, Yamaguchi A, Komori T. Multilineage differentiation of Cbfa1-deficient calvarial cells *in vitro*. Biochem Biophys Res Commun 2000; 273: 630–636. 10873656 10.1006/bbrc.2000.2981

[bib39] 39Bennett CN, Longo KA, Wright WS, Suva LJ, Lane TF, Hankenson KD et al. Regulation of osteoblastogenesis and bone mass by Wnt10b. Proc Natl Acad Sci USA 2005; 102: 3324–3329. 15728361 10.1073/pnas.0408742102 PMC552924

[bib40] 40Clement-Lacroix P, Ai M, Morvan F, Roman-Roman S, Vayssiere B, Belleville C et al. Lrp5-independent activation of Wnt signaling by lithium chloride increases bone formation and bone mass in mice. Proc Natl Acad Sci USA 2005; 102: 17406–17411. 16293698 10.1073/pnas.0505259102 PMC1297659

[bib41] 41Taylor-Jones JM, McGehee RE, Rando TA, Lecka-Czernik B, Lipschitz DA, Peterson CA. Activation of an adipogenic program in adult myoblasts with age. Mech Ageing Dev 2002; 123: 649–661. 11850028 10.1016/s0047-6374(01)00411-0

[bib42] 42Arango NA, Szotek PP, Manganaro TF, Oliva E, Donahoe PK, Teixeira J. Conditional deletion of beta-catenin in the mesenchyme of the developing mouse uterus results in a switch to adipogenesis in the myometrium. Dev Biol 2005; 288: 276–283. 16256976 10.1016/j.ydbio.2005.09.045

[bib43] 43Hooper JE, Scott MP. Communicating with Hedgehogs. Nat Rev Mol Cell Biol 2005; 6: 306–317. 15803137 10.1038/nrm1622

[bib44] 44Spinella-Jaegle S, Rawadi G, Kawai S, Gallea S, Faucheu C, Mollat P et al. Sonic hedgehog increases the commitment of pluripotent mesenchymal cells into the osteoblastic lineage and abolishes adipocytic differentiation. J Cell Sci 2001; 114: 2085–2094. 11493644 10.1242/jcs.114.11.2085

[bib45] 45Zehentner BK, Leser U, Burtscher H. BMP-2 and sonic hedgehog have contrary effects on adipocyte-like differentiation of C3H10T1/2 cells. DNA Cell Biol 2000; 19: 275–281. 10855794 10.1089/10445490050021186

[bib46] 46Suh JM, Gao X, McKay J, McKay R, Salo Z, Graff JM. Hedgehog signaling plays a conserved role in inhibiting fat formation. Cell Metab 2006; 3: 25–34. 16399502 10.1016/j.cmet.2005.11.012

[bib47] 47Simonsen JL, Rosada C, Serakinci N, Justesen J, Stenderup K, Rattan SI et al. Telomerase expression extends the proliferative life-span and maintains the osteogenic potential of human bone marrow stromal cells. Nat Biotechnol 2002; 20: 592–596. 12042863 10.1038/nbt0602-592

[bib48] 48Abdallah BM, Haack-Sorensen M, Burns JS, Elsnab B, Jakob F, Hokland P et al. Maintenance of differentiation potential of human bone marrow mesenchymal stem cells immortalized by human telomerase reverse transcriptase gene despite [corrected] extensive proliferation. Biochem Biophys Res Commun 2005; 326: 527–538. 15596132 10.1016/j.bbrc.2004.11.059

[bib49] 49Al-Nbaheen M, Vishnubalaji R, Ali D, Bouslimi A, Al-Jassir F, Megges M et al. Human stromal (mesenchymal) stem cells from bone marrow, adipose tissue and skin exhibit differences in molecular phenotype and differentiation potential. Stem Cell Rev 2013; 9: 32–43. 22529014 10.1007/s12015-012-9365-8 PMC3563956

[bib50] 50Hui AB, Shi W, Boutros PC, Miller N, Pintilie M, Fyles T et al. Robust global micro-RNA profiling with formalin-fixed paraffin-embedded breast cancer tissues. Lab Invest 2009; 89: 597–606. 19290006 10.1038/labinvest.2009.12

[bib51] 51Livak KJ, Schmittgen TD. Analysis of relative gene expression data using real-time quantitative PCR and the 2(-Delta Delta C(T)) method. Methods 2001; 25: 402–408. 11846609 10.1006/meth.2001.1262

[bib52] 52Alajez NM, Shi W, Hui AB, Yue S, Ng R, Lo KW et al. Targeted depletion of BMI1 sensitizes tumor cells to P53-mediated apoptosis in response to radiation therapy. Cell Death Differ 2009; 16: 1469–1479. 19575017 10.1038/cdd.2009.85

[bib53] 53Al-toub M, Almusa A, Almajed M, Al-Nbaheen M, Kassem M, Aldahmash A et al. Pleiotropic effects of cancer cells' secreted factors on human Stromal (mesenchymal) stem cell. Stem Cell Res Ther 2013; 4: 114. 24405819 10.1186/scrt325 PMC3854757

[bib54] 54Vishnubalaji R, Manikandan M, Al-Nbaheen M, Kadalmani B, Aldahmash A, Alajez NM. *In vitro* differentiation of human skin-derived multipotent stromal cells into putative endothelial-like cells. BMC Dev Biol 2012; 12: 7. 22280443 10.1186/1471-213X-12-7 PMC3280173

