# Supplementary material for: microRNA-320/RUNX2 axis regulates adipocytic differentiation of human mesenchymal (skeletal) stem cells
Source: Cell Death Dis. 2014 Oct 30;5(10):e1499–. doi: 10.1038/cddis.2014.462 (PMC4237271; doi:10.1038/cddis.2014.462)
Supplement: Supplementary Figure 1 [file cddis2014462x1.docx]

**Supplementary figure 1.** Workflow outline for the microRNA microarray experiment
